# A district-based health facility assessment for maternal and newborn care across Pakistan

**DOI:** 10.7189/jogh.16.04103

**Published:** 2026-04-30

**Authors:** Muhammad Ayaz Mustufa, Shabina Ariff, Nimra Shahid, Fareeha Javaid, Faiz Ahmed Raza, Uzair Ansari, Sajid Soofi, Aamer Ikram, Imran Ahmed Chauhadry, Zulfiqar Ahmed Bhutta

**Affiliations:** 1 National Institute of Health, Ministry of National Health Services, Regulations & Coordination, Islamabad, Pakistan; 2 Department of Pediatrics & Child Health, Aga Khan University, Karachi, Pakistan; 3 Centre of Excellence in Women and Child Health, Aga Khan University, Karachi, Pakistan; 4 Institute for Global Health & Development, Aga Khan University, Karachi, Pakistan; 5 Centre for Global Child Health, The Hospital for Sick Children (SickKids), Toronto, Canada

## Abstract

**Background:**

The shift from home to facility births has not translated into substantial mortality reduction in Pakistan. Therefore, we assessed whether health care facilities (HCFs) offer or have the capacity to offer critical maternal and newborn interventions. We aimed to propose tailored facility-based maternal and newborn care strategies, especially for the perinatal period.

**Methods:**

We conducted an HCF assessment in 275 public and private HCFs across 141 districts of Pakistan. We employed a comprehensive, quantitative tool to assess the availability and readiness of resources needed for critical maternal and newborn interventions, including Basic Emergency Obstetric and Newborn Care, Comprehensive Emergency Obstetric and Newborn Care signal functions, and Every Newborn Action Plan interventions. We analysed the data using Stata, version 13.0.

**Results:**

A high proportion of facilities offered delivery services (93%), yet lacked specialised staff, especially paediatric surgeons (18%) and neonatologists (18.5%). Availability of neonatal nurses was also critically low, especially in Balochistan (19.4%) and Gilgit-Baltistan (22.2%). Clinical guidelines, especially for newborn resuscitation (8.8%), kangaroo mother care (KMC) (8.4%), and use of injectable antibiotics (10.9%), were largely unavailable. Notable regional disparities were observed, with Balochistan displaying the poorest service availability and readiness. Only 8.3% of facilities in Balochistan had newborn resuscitation equipment, and none had guidelines.

**Conclusions:**

To make a substantial impact on reducing maternal and newborn mortality, data-driven, equitable allocation of funds and resources to HCFs is crucial. The availability of skilled health care providers, basic newborn care, and low-cost, high-impact interventions, such as the optimal use of antenatal corticosteroids, KMC, newborn resuscitation, and injectable antibiotics, should be prioritised and scaled up.

Neonatal mortality, defined as death within the first 28 days of life, constitutes 41% deaths in children under five [1]. Most of these deaths occur within the first week of life, at home, and in low-resource settings where health system access and referral mechanisms are weak [2–4].

Pakistan reflects this global burden, with a neonatal mortality rate (NMR) of 40 deaths per 1000 live births, which is higher than many countries with comparable economies [5]. Despite committing to the Every Newborn Action Plan’s target of reducing NMR to ten deaths per 1000 births [6], the country continues to face persistent implementation challenges, especially in remote and rural areas, where health care inequities drive preventable mortality [7].

To meet the Every Newborn Action Plan targets and close the gap between policy and implementation, it is critical to strengthen facility-based newborn care through coordinated efforts. This includes ensuring the availability of skilled health care providers capable of managing delivery complications and delivering essential newborn interventions [8]. The leading causes of neonatal mortality – prematurity (28%), severe infections (26%), and birth asphyxia (23%) [9] – are all conditions that can be significantly reduced with timely and quality facility-based care.

During the antenatal period, the use of antenatal corticosteroids (ACSs) between 24 and 34 weeks of gestation for suspected preterm birth can significantly improve foetal lung maturation and reduce the risk of respiratory distress syndrome and mortality by approximately 40% [10]. If a newborn is born prematurely but is stable at birth, facility-based kangaroo mother care (KMC), a low-cost, high-impact intervention, can lower mortality by 51% [11], especially in those weighing <2000 g [12,13].

Intrapartum complications, such as birth asphyxia and intrauterine hypoxia, require immediate newborn resuscitation, yet coverage remains low. If 90% of facility-based births included effective newborn resuscitation, an estimated 93 000 neonatal deaths could be prevented each year [14]. Similarly, administering injectable antibiotics during this period can avert nearly one-third of neonatal mortality [15].

Maternal interventions include the management of pre-eclampsia/eclampsia and postpartum haemorrhage (PPH), which account for 16% and 27% of maternal mortality, respectively [16]. Critical interventions to manage these complications include Basic Emergency Obstetric and Newborn Care (BEmONC) and Comprehensive Emergency Obstetric and Newborn Care (CEmONC) signal functions. BEmONC signal functions include administering maternal parenteral antibiotics, intravenous infusion/fluid replacement therapy, manual uterine exploration and removal of placenta, removal of retained products of conception, assisted vaginal birth (*e.g.* vacuum extractor, forceps); while CEmONC signal functions include all aforementioned interventions, blood transfusion, and caesarean section.

Since the provision of facility-based care relies on resource availability and service readiness, we conducted a comprehensive national-level survey, the Maternal and Newborn Health Facility Assessment (MNHFA), in 2022. This assessment evaluated whether large secondary (*i.e.* district headquarter hospitals (DHQHs)) and, where unavailable, tertiary health care facilities (HCFs) had the resources and capacity to provide secondary maternal and newborn care.

## METHODS

### Study design

The MNHFA was a cross-sectional, HCF assessment we conducted in 2022 across Pakistan. The survey employed a comprehensive and structured quantitative tool. This tool was developed by integrating the World Health Organization’s Service Availability and Readiness Assessment tool [17], the World Bank’s Service Delivery Indicators tool [18], and the United Nations Population Fund’s Service Provision Assessment tool [19].

We analysed the availability and readiness of critical maternal and newborn interventions, as per the World Health Organization’s Service Availability and Readiness Assessment [17]. Key indicators of service availability include an adequate health workforce and essential infrastructure, while key indicators of service readiness include infection prevention measures, clinical guidelines, trained staff, and the essential medicines and equipment required to deliver critical interventions.

The critical interventions we assessed include the high-impact interventions of the Every Newborn Action Plan, BEmONC, and CEmONC signal functions [20]. These interventions address high-mortality complications, including pre-eclampsia/eclampsia, PPH, severe infections, birth asphyxia, and preterm birth – leading causes of maternal and newborn mortality (Table S1 in the **Online Supplementary Document**).

### Sample

We assessed a total of 275 public and private HCFs across all four provinces of Pakistan: Sindh, Punjab, Balochistan, and Khyber Pakhtunkhwa (KP), as well as the administrative territories of Azad Jammu and Kashmir (AJK) and Gilgit-Baltistan (GB). We collected a representative sample of public HCFs by stratifying and mapping districts across the country, selecting 141 districts. Since the MNHFA focused on secondary newborn care, we primarily included secondary HCFs, referred to as DHQHs. However, in districts where DHQHs were either unavailable or non-functional, we surveyed tertiary care or specialised teaching hospitals.

To ensure representation from the private sector, given its role in caring for a significant portion of the country's population, we drew approximately 45% of the sample from private maternity and newborn facilities. As private facilities are not structured within the same administrative hierarchy as public HCFs, we selected the sample using purposive sampling based on their service scope. These included maternity clinics, medical centres, medical complexes, and maternity homes (**Table 1**). We excluded primary HCFs from the survey since they are not mandated to offer secondary newborn care (Table S2 in the **Online Supplementary Document**).

**Table 1:** Description of the sample, n (%)

	Total	AJK	Balochistan	GB	KP	Punjab	Sindh
**Total number of HCFs**	275	19	36	19	58	85	58
**HCF type**							
Private	124 (45.1)	8 (42.1)	8 (22.2)	7 (36.8)	28 (48.3)	42 (49.4)	31 (53.4)
Public	151 (54.9)	11 (57.9)	28 (77.8)	12 (63.2)	30 (51.7)	43 (50.6)	27 (46.6)
**Setting**							
Urban	204 (74.2)	12 (63.2)	16 (44.4)	11(57.9)	50 (86.2)	79 (92.9)	36 (62.1)
Rural	71 (25.8)	7 (36.8)	20 (55.6)	8 (42.1)	8 (13.8)	6 (7.1)	22 (37.9)
**HCF level**							
DHQH	120 (43.6)	8 (42.1)	28 (77.8)	8 (42.1)	26 (44.8)	31 (36.5)	19 (32.8)
Specialised teaching institutes	14 (5.1)	3 (15.8)	0 (0.0)	0 (0.0)	1 (1.7)	8 (9.4)	2 (3.4)
Tertiary care hospitals	45 (16.4)	0 (0.0)	1 (2.8)	2 (10.5)	10 (17.2)	14 (16.5)	18 (31.0)
Others	96 (34.9)	8 (42.1)	7 (19.4)	9 (47.4)	21 (36.2)	32 (37.6)	19 (32.8)
**Managing authority of HCF**							
Government/public	152 (55.3)	11 (57.9)	27 (75.0)	12 (63.2)	32 (55.2)	42 (49.4)	28 (48.3)
NGO/not-for-profit	21 (7.6)	0 (0.0)	0 (0.0)	1 (5.3)	4 (6.9)	10 (11.8)	6 (10.3)
Private for-profit	98 (35.6)	8 (42.1)	8 (22.2)	5 (26.3)	22 (37.9)	33 (38.8)	22 (37.9)
Public-private partnership	4 (1.5)	0 (0.0)	1 (2.8)	1 (5.3)	0 (0.0)	0 (0.0)	2 (3.4)

AJK – Azad Jammu and Kashmir, DHQH – district headquarter hospital, GB – Gilgit Baltistan, HCF – health care facility, KP – Khyber Pakhtunkhwa, NGO – non-governmental organisation

### Data collection and analysis

Survey staff were specifically trained in the use of survey tools, and we conducted regular quality assurance checks. We also used a detailed survey tool and booklet for internal and external validation and to ensure harmonisation at each step of the survey (Table S3 and Appendix S1 in the **Online Supplementary Document**).

Some denominators varied across indicators due to missing data. We analysed the available data and explicitly reported denominators when they differed from the total sample size. We conducted a descriptive analysis of key variables to assess service availability and readiness of HCFs across Pakistan.

We used Stata, version 13.0 (StataCorp LLC, College Station, Texas, USA) for all analyses.

## RESULTS

### Infrastructural availability

**Figure 1:**
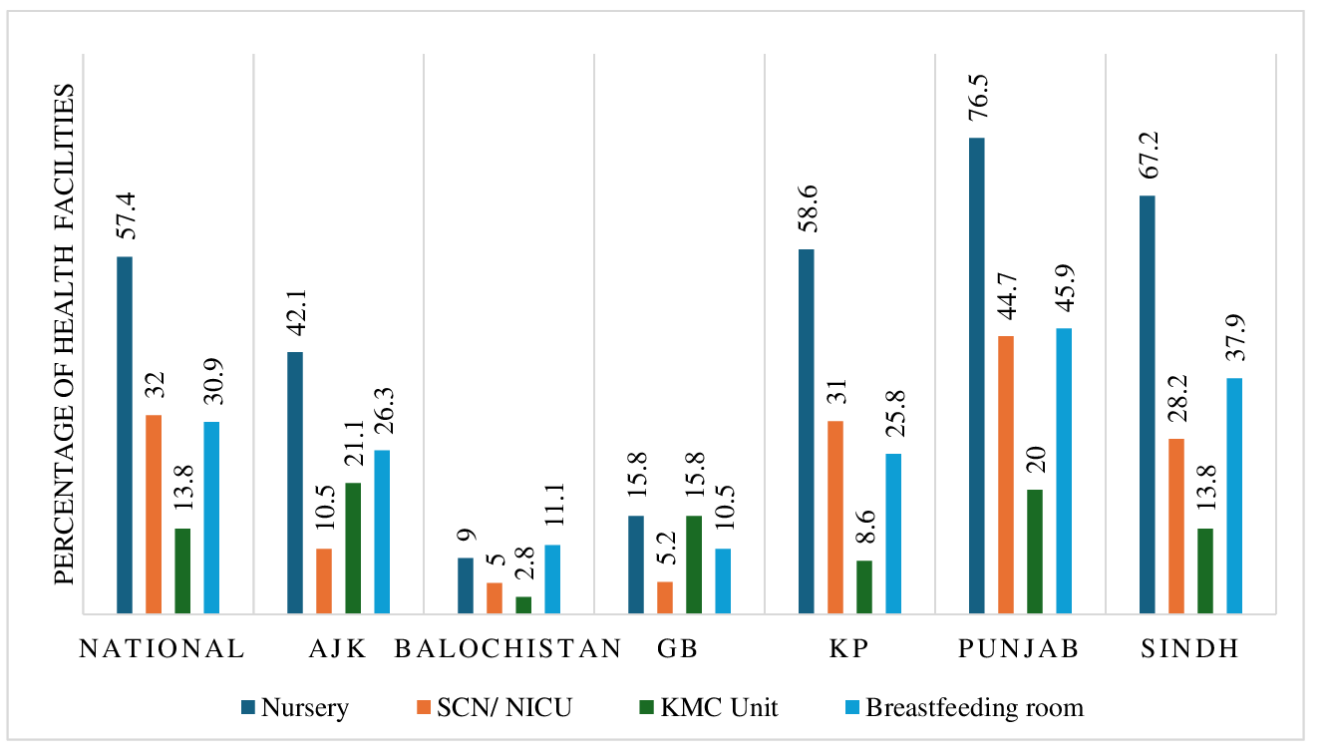
Distribution of newborn relevant infrastructure and services available across provinces. AJK – Azad Jammu and Kashmir, GB – Gilgit Baltistan, KP – Khyber Pakhtunkhwa, SCN – special care nursery, NICU – neonatal intensive care unit, KMC – kangaroo mother care.

Infrastructure for newborn care, including care for small and sick newborns, encompasses nurseries, special care nurseries, neonatal intensive care units (NICUs), KMC units, and baby-friendly breastfeeding rooms. Of the 275 HCFs assessed, 57.4% had nurseries, and 32.0% had a special care nursery or NICU (**Figure 1**). For feeding support, we assessed the availability of baby-friendly/breastfeeding rooms, which were present in 30.9% of the sampled facilities. For KMC, we assessed the availability of KMC units, which were present in 13.8% facilities.

Among all regions, Punjab exhibited the highest infrastructural capacity, with 76.5% of facilities equipped with nurseries. In contrast, Balochistan showed the lowest capacity, with 11% of facilities having breastfeeding rooms, 9% having nurseries, 5% having NICUs, and 1% having KMC units. Similarly, in GB, 5.2% of facilities had NICUs, 10.5% had breastfeeding rooms, and 15.8% had nurseries, and 15.8% had KMC units.

### Availability of specialised health workforce

We assessed the availability of key health care cadres, including nurses, neonatologists, paediatricians, paediatric surgeons, gynaecologists, and obstetricians, and identified significant human resource gaps and marked regional disparities. Workforce availability was defined as the total number of staff working at the facility at the time of the survey, regardless of shift patterns, full-time or part-time status, or continuous (*i.e.* 24/7) presence in newborn care areas.

**Figure 2:**
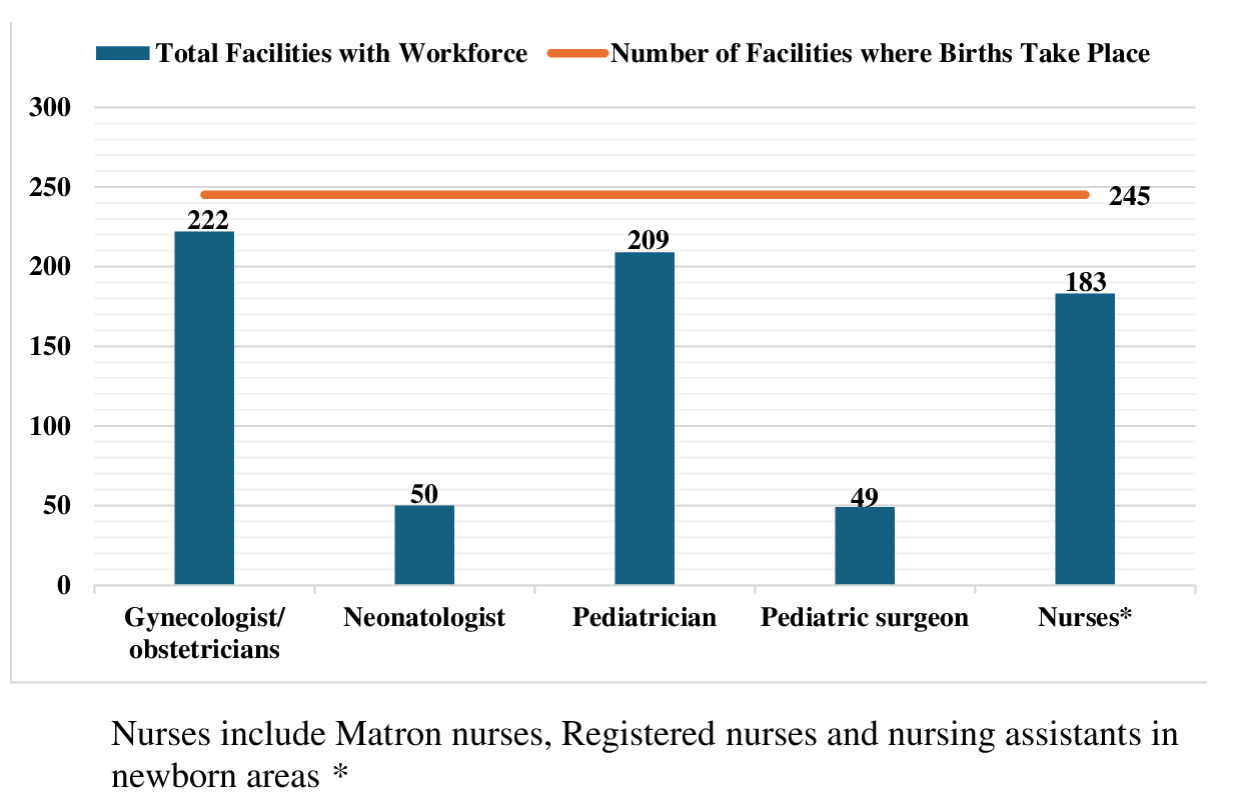
Facilities and MNCH cadre across provinces. MNCH – maternal, newborn, and child health.

Of 275 HCFs, paediatric surgeons were available in 49 (18%) and neonatologists in 50 (18.5%) facilities (**Figure 2**). To evaluate the alignment between staffing and service delivery, we compared human resource availability with the proportion of facilities that offer delivery services. We found critical service delivery gaps, with many facilities providing maternal and newborn care in the absence of essential staff. A significant proportion of facilities offered delivery services but lacked nurses (n = 63), paediatric surgeons (n = 196), or neonatologists (n = 195) for immediate newborn care.

Regional disparity was observed in nurse availability, with 86.9% of facilities in Punjab having nurses, compared to only 19.4% in Balochistan and 22.2% in GB (Table S4 in the **Online Supplementary Document**). Neonatologist availability was even more limited, which were present in 2.8% of facilities in Balochistan and 11% of facilities in GB. Notably, there were no paediatric surgeons in GB and no neonatologists in AJK.

### Care during pregnancy and childbirth

Key interventions for care during pregnancy and childbirth include management of pre-eclampsia/eclampsia and PPH, as they are leading causes of maternal mortality. While magnesium sulphate for pre-eclampsia management was found in 73.5% of facilities, guidelines, in the form of folders or wall mounts, were observed in only 21.9% of facilities (**Table 2**). Similarly, oxytocin for PPH management was found in 83.3% facilities, but guidelines were observed in only 17.9%. Availability of ACS (57.7%) and guidelines on the use of ACS for foetal lung maturation (10.2%) were comparatively low.

**Table 2:** Distribution of facilities with guidelines and trained staff to offer maternal services*

	Total	AJK	Balochistan	GB	KP	Punjab	Sindh
**Total HCFs**	275	19	36	19	58	85	58
**Management of preterm birth**							
Availability of guidelines	28 (10.2)	3 (15.8)	3 (8.3)	1 (5.6)	3 (5.2)	8 (9.4)	10 (17.2)
Availability of ACSs	158/274 (57.7)	14 (73.7)	8 (22.2)	7/18 (38.9)	37(63.8)	59 (69.4)	33 (56.9)
Availability of services	178 (64.7)	15 (78.9)	14 (38.9)	5 (26.3)	38 (65.5)	63 (74.1)	43 (74.1)
**Skilled birth care**							
Guidelines on prevention of PPH	49 (17.9)	4 (21.1)	5 (13.9)	0 (0.0)	4 (6.9)	20 (23.5)	16 (27.6)
Availability of oxytocin	229 (83.3)	19 (100.0)	19 (52.8)	14 (73.7)	51 (87.9)	73 (85.9)	53 (91.4)
**BEmONC**							
Guidelines on management of pre-eclampsia	60 (21.9)	6 (31.6)	4 (11.1)	2 (11.1)	8 (13.8)	24 (28.2)	16 (27.6)
Availability of magnesium sulphate	202 (73.5)	18 (94.7)	15 (41.7)	9 (47.4)	44 (75.9)	69 (81.2)	47 (81.0)
Availability of assisted vaginal birth and its equipment	164 (59.6)	11 (57.9)	10 (27.8)	7 (36.8)	40 (69.0)	52 (61.2)	44 (75.9)
**CEmONC**							
Obstetric surgery anaesthesia	164 (59.6)	14 (73.7)	7 (19.4)	5 (26.3)	36 (62.1)	63 (74.1)	39 (67.2)
Blood bank and transfusion by trained staff	58 (21.1)	0 (0.0)	2 (5.6)	0 (0.0)	12 (20.7)	27 (31.8)	17 (29.3)
Blood bank or transfusion by trained staff	184 (66.9)	8 (42.1)	10 (27.8)	5 (26.3)	47 (81.0)	72 (84.7)	42 (72.4)

ACS – antenatal corticosteroid, AJK – Azad Jammu and Kashmir, BEmONC – Basic Emergency Obstetric and Newborn Care, CEmONC – Comprehensive Emergency Obstetric and Newborn Care, GB – Gilgit Baltistan, HCF – health care facility, KP – Khyber Pakhtunkhwa, NGO – non-governmental organisation, PPH – postpartum haemorrhage

*All values are presented as numbers (percentages), denominators are explicitly stated when they differ from the total sample size.

In terms of service delivery, around two-thirds of the facilities had the equipment (*i.e.* functional vacuum extractors and delivery instruments) for assisted vaginal deliveries (59.6%), infrastructure for obstetric surgeries (59.6%), and either blood bank services or trained staff (66.9%). AJK had the best-equipped HCFs, with high availability of medicines such as ACS (73.7%), oxytocin (100%), and magnesium sulphate (94.7%), but poor availability of guidelines and no HCF with both a blood bank and trained staff for blood transfusion. Sindh and Punjab had the highest proportion of facilities with guidelines.

Of the 275 facilities, only one public HCF had all 11 maternal signal functions. Many public facilities (n = 31) offered the majority of the signal functions (8–10) compared to private facilities (n = 17) (Figures S1 and S2 in the **Online Supplementary Document**).

### Immediate newborn care

We assessed whether HCFs were equipped with the necessary guidelines, trained staff, essential medicines, and equipment to deliver immediate newborn care. Key immediate newborn care interventions include basic newborn care, KMC, newborn resuscitation, cord care, thermal protection, and feeding support.

We found that 54.7% of facilities had staff trained in basic newborn care (**Table 3**), with the lowest proportions reported in Balochistan (25%) and GB (22.2%). Indicators were most promising for cord care, with 67.5% of facilities having guidelines for delayed cord clamping and 89.1% stocking chlorhexidine. While more than two-thirds of facilities had warmers (70.8%) and incubators (69.3%) for thermal management, only 9.9% had corresponding guidelines. Indicators for feeding support were the weakest, as guidelines were available in 19.3% of facilities and appropriate equipment in 10.6% of facilities.

**Table 3:** Distribution of facilities with guidelines and trained staff to offer Immediate Newborn Care*

	Total	AJK	Balochistan	GB	KP	Punjab	Sindh
**Total number of HCFs**	274	19	36	18	58	85	58
**Newborn resuscitation**							
Training	117/262 (44.7)	4/17 (23.5)	3/35 (8.6)	5/17 (29.4)	21/53 (39.6)	51/82 (62.2)	33 (56.9)
Guidelines	24 (8.8)	4 (21.1)	0 (0.0)	0 (0.0)	1 (1.7)	8 (9.4)	11 (19.0)
Bag and mask	183 (66.8)	14 (73.7)	11 (30.6)	8 (44.4)	34 (58.6)	72 (84.7)	44 (75.9)
Crash cart†	153 (55.8)	10 (52.6)	3 (8.3)	3 (16.7)	31 (53.4)	61 (71.8)	45 (77.6)
**BNC**							
Training on BNC	150 (54.7)	8 (42.1)	9 (25.0)	4 (22.2)	38 (65.5)	53 (62.4)	38 (65.5)
Guideline on thermal management	27 (9.9)	3 (15.8)	2 (5.6)	0 (0.0)	0 (0.0)	12 (14.1)	10 (17.2)
Warmer	194 (70.8)	15 (78.9)	16 (44.4)	10 (55.6)	40 (69.0)	72 (84.7)	41 (70.7)
Incubator	190 (69.3)	13 (68.4)	15 (41.7)	8 (44.4)	38 (65.5)	71 (83.5)	45 (77.6)
Guideline on delayed cord clamping	185 (67.5)	15 (78.9)	10 (27.8)	12 (66.7)	49 (84.5)	50 (58.8)	49 (84.5)
Guideline on hygienic cord care	244 (89.1)	18 (94.7)	27 (75.0)	16 (88.9)	52 (89.7)	77 (90.6)	54 (93.1)
Chlorhexidine	244 (89.1)	18 (94.7)	27 (75.0)	16 (88.9)	52 (89.7)	77 (90.6)	54 (93.1)
Guideline on newborn feeding	53 (19.3)	4 (21.1)	3 (8.3)	0 (0.0)	8 (13.8)	11 (12.9)	27 (46.6)
At least 3 feeding support	29 (10.6)	7 (8.2)	16 (27.6)	1 (2.8)	4 (6.9)	1 (5.6)	0 (0.0)
**KMC**							
Training	62 (22.6)	5 (26.3)	1 (2.8)	2 (11.1)	24 (41.4)	19 (22.4)	11 (19.0)
Guidelines	23 (8.4)	3 (15.8)	2 (5.6)	0 (0.0)	2 (3.4)	10 (11.8)	6 (10.3)
KMC beds	33 (12.0)	5 (26.3)	1 (2.8)	0 (0.0)	5 (8.6)	13 (15.3)	9 (15.5)

AJK – Azad Jammu and Kashmir, BNC – basic newborn care, GB – Gilgit Baltistan, HCF – health care facility, KMC – kangaroo mother care, KP – Khyber Pakhtunkhwa

*All values are presented as n (%), denominators are explicitly stated when they differ from the total sample size.

†Crash cart refers to a functional resuscitation area with crash cart in newborn area.

The availability of newborn resuscitation bags with size-appropriate masks (66.8%) and a functional resuscitation area (55.8%) was generally strong. Sindh (77.6%) and Punjab (71.8%) performed notably well, whereas Balochistan (8.3%) and GB (16.7%) lagged significantly. On the contrary, guidelines for newborn resuscitation were only observed in 8.8% of facilities, with none found in Balochistan or GB. The proportion of trained staff was moderate at 44.7% nationwide. Punjab (62.2%) and Sindh (56.9%) showed promising levels, while Balochistan (8.6%) and AJK (23.5%) lagged.

Overall, the availability of guidelines for KMC was low (8.4%), with the highest in AJK (15.8%) and Punjab (11.8%), and lowest in GB (0%), followed by KP (3.4%) and Balochistan (5.6%). The presence of trained staff was comparatively better (22.6%), particularly in KP (41.4%) and Punjab (22.4%). Beds were available in only 12% of facilities, with none in GB and only 2.8% in Balochistan.

Only three public and one private HCFs offered the most (8–10) newborn signal functions. More public facilities (n = 24) offered 6–8 signal functions than private facilities (n = 8) (Figures S3 and S4 in the **Online Supplementary Document**).

### Infection prevention and management

The availability of guidelines for the use of injectable antibiotics remained low at 10.9%. Sindh had relatively better guideline availability (22.4%), whereas GB had none. Injectable antibiotics needed to treat severe infections were unavailable in over half of the facilities. The availability varied slightly by category. Ampicillin was available in 41.8% of facilities and gentamicin in 37.5%; these two medications are the first-line agents for protecting against newborn infections and are used universally. Surprisingly, the availability of gentamicin was low in Punjab (27.1%), while the availability of ampicillin was low in Balochistan (16.7%), highlighting issues in contextual knowledge and supply chain.

Guidelines on standard precautions for infection prevention were observed in only a third of facilities (32.5%), with the highest proportions in Sindh (19.0%) and AJK (21.1%), and the lowest in Balochistan (8.3%) and KP (10.3%). More than two-thirds of the facilities had supplies like clean water, soap, gloves, syringes, sharps containers, and disinfectants for infection prevention. However, in KP, less than half of the facilities had alcohol-based hand rubs (25.9%) and environmental disinfectants (39.7%). Clean running water (94.5%) and disposable syringes (93.8%) were most readily available, while alcohol-based hand rub (62.0%) and separate bins for clinical and non-clinical waste (64.6%) were least readily available (Table S5 in the **Online Supplementary Document**).

## DISCUSSION

The percentage of live births delivered in HCFs increased from 34% in 2006–2007 to 66% 2017–2018 [21]. However, this increased service utilisation and shift to facility births have not translated into improvements in newborn care and health outcomes, indicating the need to assess whether HCFs are equipped to provide critical maternal and newborn care interventions and manage common complications that result in mortality.

We evaluated the service availability and readiness of key maternal and newborn interventions in 275 public and private HCFs across Pakistan. These key interventions include ACS, newborn resuscitation, KMC, and injectable antibiotics, which are low-cost and high-impact in reducing NMR. Although DHQHs need to be equipped with a broad range of secondary services, we found low service availability and readiness to deliver interventions, especially for newborn care.

Service availability was poor across Pakistan, especially for the specialised workforce (*e.g.* neonatologists, neonatal surgeons, and neonatal nurses), consistent with previous studies [22,23]. In addition to staff hiring, there is a need for formal training and refresher courses for existing staff, as a study assessing the basic knowledge of medical officers in providing maternal and newborn care found that only a third scored above the minimum competency level [23]. Prioritising investments in NICUs and special care baby units is also essential to strengthen service availability and improve newborn outcomes [24].

We also found a stark disparity across regions, with results comparatively better in Punjab and Sindh but poor in Balochistan, GB, and AJK. For instance, while trained staff in newborn resuscitation were present in 44.7% facilities in Pakistan, coverage varied dramatically. Punjab reported the highest availability (62.2%), while Balochistan reported the lowest (8.6%). Equipment availability showed a similar trend. Although 66.8% of facilities had newborn resuscitation bags and 55.8% had functional resuscitation areas, regional gaps persisted, with Sindh being well-equipped (77.6%) and Balochistan having the lowest availability (8.3%). This reflects poor and inequitable resource allocation, which may be linked with the high mortality rates in Balochistan.

Balochistan, being the largest province, with the highest NMR of 63 deaths per 1000 live births [24], drew concern. We found limited resource availability and low infrastructural and human resource capacity in Balochistan HCFs, consistent with previous studies [25,26]. The Pakistan Demographic and Health Survey 2017–2018 also provides evidence of poor service utilisation and health-seeking behaviour, reporting that 40% of women who had given birth in the five years preceding the survey did not receive any antenatal care. Furthermore, only 36.1% of newborns received a postnatal check-up within 42 hours of birth, indicating critical gaps.

The disparity and poor service availability and readiness can be linked to the distribution of financial resources. Despite the country's high burden of maternal and newborn mortality, only 3% of the national health budget (*i.e.* PKR 17 753 million) is allocated to maternal and newborn health, with the smallest shares directed to AJK, GB, and Balochistan [27]. This underinvestment stems from Pakistan’s continued reliance on population-based funding formulas, which fail to account for regional disparities in health needs. A utilisation-incidence analysis revealed that in 2007–2008, Balochistan received just 5.4% of the national health budget, matching its population share but ignoring its disproportionately poor health indicators and geographic challenges [28]. Addressing these financing constraints and systemic inequities is essential for improving service readiness and reducing newborn mortality in the most underserved regions.

### Limitations and future recommendations

This study has some limitations. The cross-sectional design captures facility readiness at a single point in time. As the analysis was limited to descriptive statistics, we could not draw any inferential conclusions, and findings therefore reflect only patterns observed within the sampled facilities. Future research should include further statistical analysis of the MNHFA data set to draw inferences.

Since private HCFs do not follow the same categorisation as public facilities, randomisation was not feasible, so we employed a purposive sampling technique based on their mandate. This may have introduced selection bias and limits representativeness and generalisability of findings across all private HCFs.

Workforce availability was measured based on nominal staffing levels at the time of assessment and did not capture cadre-specific deployment, shift coverage, or continuous availability in newborn units. As such, the reported presence of specialised personnel may overestimate functional readiness, particularly during off-hours or peak service demand.

Awareness of the assessment purpose by facility staff may have introduced a Hawthorne effect, potentially leading to overreporting of service availability or functionality. Furthermore, several indicators relied on self-reported information, including the availability and functionality of equipment, adherence to guidelines, and staff training, which may be subject to reporting bias or misclassification.

We could not directly observe clinical practice or conduct retrospective case reviews to assess the quality of care for mothers and newborns across facilities. However, it was evident that many breastfeeding rooms and KMC bays were not implementing the interventions despite conducive environments and prior training. Future evaluations should also include elements of policy analysis, service delivery, and district health information systems to further enrich and triangulate the findings.

## CONCLUSIONS

Pakistan has had a consistently high burden of maternal and newborn mortality. Most of these deaths are largely due to preventable causes and can be prevented with high-impact, low-cost interventions, including BEmONC and CEmONC signal functions. We found gaps in the availability and readiness of HCFs to deliver these critical interventions, which can contribute to the poor mortality indicators.

Addressing these gaps is crucial and requires coordinated actions across multiple levels of the health system. At the facility level, priority should be given to ensuring the consistent availability of guidelines, resources, and trained staff. At the district and provincial level, a robust monitoring and health information system is essential to prevent stock-outs and ensure data-driven, need-based resource allocation. Lastly, at the national level, guidelines should be regularly updated, disseminated, and integrated into pre-service and in-service training curricula. Ensuring alignment between policy, financing, and service delivery is also essential to improve maternal and neonatal health outcomes.

## Additional material


Online Supplementary Document
