# Supplementary material for: A district-based health facility assessment for maternal and newborn care across Pakistan
Source: J Glob Health. 2026 Apr 30;16:04103. doi: 10.7189/jogh.16.04103 (PMC13132009; doi:10.7189/jogh.16.04103)
Supplement: Online Supplementary Document [file jogh-16-04103-s001.pdf]

Supplement to: Mustufa MA, Ariff S, Shahid N, Javaid F, Raza FA, Ansari U, Soofi S, Ikram A, Chauhadry IA, Bhutta ZA. A district-based health facility assessment for maternal and newborn care across Pakistan. J Glob Health. 2026;16:04103.

Table S1. Key Interventions of the study

**Care during Pregnancy and Childbirth**	**Management of pre-term birth**<br>**Skilled care at birth-** use of clean birth kit, oxytocin<br>**Basic Emergency Obstetric Care** - Assisted vaginal delivery; parenteral magnesium sulfate for (pre-)eclampsia<br>**Comprehensive Emergency Obstetric Care-** Surgery (e.g., caesarean section) including anesthesia; blood transfusion
**Immediate Newborn Care**	**Basic Newborn Care**<br>• **Hygienic cord care** (cutting with sterile blade, application of 4% chlorhexidine on tip of the cord and stump and no application of harmful substances)<br>• **Thermal protection**<br>• **Immediate and exclusive breastfeeding**<br>**Kangaroo Mother Care (KMC)**<br>**Newborn resuscitation**

Table S2. Description of Sample

Province	Total<br>n (%)	AJK<br>n (%)	Balochistan<br>n (%)	GB<br>n (%)	KP<br>n (%)	Punjab<br>n (%)	Sindh<br>n (%)
**Total Number of HCFs (N)**	**275**	**19**	**36**	**19**	**58**	**85**	**58**
**Health Facility type**							
Private	124 (45.1)	8 (42.1)	8 (22.2)	7 (36.8)	28 (48.3)	42 (49.4)	31 (53.4)
Public	151 (54.9)	11 (57.9)	28 (77.8)	12 (63.2)	30 (51.7)	43 (50.6)	27 (46.6)
**Setting**							
Urban	204 (74.2)	12 (63.2)	16 (44.4)	11(57.9)	50 (86.2)	79(92.9)	36(62.1)
Rural	71 (25.8)	7 (36.8)	20 (55.6)	8 (42.1)	8 (13.8)	6 (7.1)	22 (37.9)
**Health Facility Level**							
District Head Quarter Hospital	120 (43.6)	8 (42.1)	28 (77.8)	8 (42.1)	26(44.8)	31 (36.5)	19 (32.8)
Specialized teaching institutes	14 (5.1)	3 (15.8)	0 (0.0)	0 (0.0)	1 (1.7)	8 (9.4)	2 (3.4)
Tertiary care hospitals	45 (16.4)	0 (0.0)	1 (2.8)	2 (10.5)	10 (17.2)	14 (16.5)	18 (31.0)
Others	96 (34.9)	8 (42.1)	7 (19.4)	9 (47.4)	21 (36.2)	32 (37.6)	19 (32.8)
**Managing Authority of Health Facility**							
Government/Public	152 (55.3)	11 (57.9)	27 (75.0)	12 (63.2)	32 (55.2)	42 (49.4)	28(48.3)
NGO / Not-for-Profit	21 (7.6)	0 (0.0)	0 (0.0)	1 (5.3)	4 (6.9)	10 (11.8)	6 (10.3)
Private for Profit	98 (35.6)	8 (42.1)	8 (22.2)	5 (26.3)	22 (37.9)	33 (38.8)	22 (37.9)
Public Private Partnership	4 (1.5)	0 (0.0)	1 (2.8)	1 (5.3)	0 (0.0)	0 (0.0)	2 (3.4)

Table S3. Health Facility Assessment Tool

BASIC INFORMATION		FORM SERIAL NUMBER				
1.	Name of Data Collector					
2.	Education Level of Data Collector<br>(Write completed years of education)					
3.	Age of Data Collector (in years)					
4.	Gender of Data Collector	1. Male<br>2. Female				
5.	Date of visit (dd-mm-yyyy)					
6.	Time of visit (24 hour format)					
7.	Province					
8.	District					
9.	Taluks/Tehsil					
10.	GIS location (mention geo location points)					
11.	Health Facility type	1. Public Health Facility<br>2. Private Health Facility<br>3. Other Specify				
12.	Health Facility	1. Urban<br>2. Rural				
13.	Health Facility level	1. District Head Quarter (DHQ) Hospital<br>2. Specialized teaching institutes<br>3. Tertiary care hospitals<br>4. Others Specify				
14.	Name of Health Facility					
15.	Managing Authority of Health Facility	1. Government/Public<br>2. Government Contractual<br>3. Public Private partnership<br>4. NGO / Not for Profit<br>5. Private for Profit<br>6. Others Specify				
16.	How many days in a week this health facility is operational?	____ ____ days				

17.	How many hours in a day this health facility is Operational?	____ ____ hours

SECTION 1: SCOPE OF SERVICES		
**Details of Respondent**		
1.	Age of Respondent (in years)	
2.	Gender of Respondent	1. Male<br>2. Female
3.	What are your main responsibilities?	1. Unit manager<br>2. Facility manager<br>3. Patient care provider<br>4. Other Specify
4.	Please tell me your highest level of professional qualification.	1. MBBS<br>2. MCPS<br>3. FCPS<br>4. Diploma<br>5. MBA<br>6. BSCN<br>7. MSCN<br>8. MSC<br>9. PhD<br>10. Other Specify
5.	Years of Experience (write in years)	
6.	How long have been working in this facility (write in years)	
7.	What are your main responsibilities?	5. Unit manager<br>6. Facility manager<br>7. Patient care provider<br>8. Other Specify
**Details of Services**		
8.	Does this facility offer delivery services	1. Yes<br>2. No
9.	Which of the following maternal services are available at this facility?<br>Mark all that applies	1. Normal delivery<br>2. Assisted vaginal delivery<br>3. Caesarean section delivery<br>4. High risk delivery<br>5. Threatened preterm birth<br>6. Obstetric surgery with anesthesia<br>7. Manual removal of placenta<br>8. Removal of retained products of conception<br>9. Others (specify)
10.	Is there 24-hour staff coverage for maternal unit,<br><br>24-hours every day including weekends, to provide	1. Yes, present, duty roster observed<br>2. Yes, present, duty roster reported but not seen<br>3. Yes, on call, duty roster observed

	care?<br><br>If YES, ask to see a duty roster/call list or schedule for 24-hour staff assignment.	4. Yes, on call, duty roster reported not seen<br>5. Duty roster not maintained<br>6. No 24-hour coverage
11.	If the facility does not offer 24 hour maternal service how many hours does it provide the services<br>(Write number of hours)	
12.	Average number of deliveries per month<br>Conducted at this facility? Verify with Register	
13.	Average number of c-section per month<br>conducted at this facility? Verify with Register	
14.	Does this facility offer delivery services	1. Yes<br>2. No
15.	Which of the following newborn services are available at this facility?<br>Mark all that applies	1. Care of term healthy newborn -well baby<br>2. Care of Preterm/LBW newborn<br>3. Care of newborn on oxygen<br>4. Care of newborn for non-invasive ventilator support<br>5. Care of newborn on invasive ventilator support<br>6. Care of newborns requiring any surgical procedure<br>7. Care of newborns with congenital cardiac malformations<br>8. Others (specify)
16.	Is there 24-hour staff coverage for newborn unit?<br><br>24-hours every day, including weekends, to provide care?<br><br>If YES, ask to see a duty roster/call list or schedule for 24-hour staff assignment	1. Yes, present, duty roster observed<br>2. Yes, present, duty roster reported but not seen<br>3. Yes, on call, duty roster observed<br>4. Yes, on call, duty roster reported not seen<br>5. Duty roster not maintained<br>6. No 24-hour coverage<br>7. Others Specify
17.	If the facility does not offer 24 hour newborn service how many hours does it provide the services<br>(Write number of hours)	
18.	Total number of births in last 12 months	
19.	Number of births in the last month	
20.	Do you admit preterm babies (< 37 weeks of gestation) in the facility?	1.Yes<br>2.No
21.	What is the minimum gestational age of baby facility has received and cared for?	____ ____ Age in weeks
22.	Total Number of preterm births per month (< 37 weeks of gestation). (Last 12 months)	
23.	Number of births in the last month (< 37 weeks of gestation).	
24.	Do you admit Low birthweight babies (< 2500	1.Yes<br>2.No

	grams) in the facility?	
25.	What is the minimum weight baby facility has received and cared for?	____ ____ ____ ____ grams
26.	Total Number of Low birthweight babies (< 2500 grams). (Last 12 months)	
27.	Low birthweight as per the categories admitted to this facility. (Last 12 months)	1. Low birth weight more than 1500 and less than 2500 grams<br>____ ____<br>2. Very low birth weight (VLBW): Weight less than 1500 grams<br>____ ____<br>3. Extremely low birth weight (ELBW): weighs less than 1000 grams<br>____ ____
28.	Number of Low birthweight babies (< 2500 grams) in the last month	
29.	Low birthweight as per the categories admitted to this facility in the last month.	4. Low birth weight more than 1500 and less than 2500 grams<br>____ ____<br>5. Very low birth weight (VLBW): Weight less than 1500 grams<br>____ ____<br>6. Extremely low birth weight (ELBW): weighs less than 1000 grams<br>____ ____
30.	Which of the following ancillary services are available at this facility?<br>Mark all that applies	1. Blood Bank<br>2. Radiology<br>3. Pharmacy<br>4. Laboratory<br>5. CSD or sterilization facilities<br>6. Biomed Services<br>7. Others (specify)

**COMMENTS/REMARKS**

SECTION 2: INFRASTRUCTURE				
Details of the Respondent (Details will be required if the respondent is different from the previous section)				
1.	Age of Respondent (in years)			
2.	Gender of Respondent	1. Male<br>2. Female		
3.	What are your main responsibilities?	1. Unit manager<br>2. Facility manager<br>3. Patient care provider<br>4. Other Specify		
4.	Please tell me your highest level of professional qualification.	1. MBBS<br>2. MCPS<br>3. FCPS<br>4. Diploma<br>5. MBA<br>6. BSCN<br>7. MSCN<br>8. MSC<br>9. PhD<br>10. Other Specify		
5.	Years of Experience (write in years)			
6.	How long have been working in this facility (write in years)			
7.	What are your main responsibilities?	1. Unit manager<br>2. Facility manager<br>3. Patient care provider<br>4. Other Specify		
Details of Infrastructure (Use tick mark to record response)		Yes	No	Number
8.	Triage Room/ER/OPD Room			
9.	Pre -Labor room			
10.	Labor Room (LR)			
11.	Operating Theater (OT)			
12.	Mother-Baby unit- Rooming In			
13.	Maternal High dependency Units (HDU)			
14.	Nursery			
15.	Special Care Nursery/Neonatal ICU			
16.	Kangaroo Mother Care unit			

17.	Baby Friendly/ Breastfeeding room			
18.	Others (specify)			
	**Observe and record below questions**			
19.	Is the same bed occupied by more than one woman?			
20.	How many babies are placed in one cot?			
21.	How many babies are placed in one warmer?			
22.	How many babies are placed in an incubator			

**SECTION 3: MATERNAL SERVICES**		
**Details of the Respondent (Details will be required if the respondent is different from the previous section)**		
1.	Age of Respondent (in years)	
2.	Gender of Respondent	1. Male<br>2. Female
3.	What are your main responsibilities?	1. Unit manager<br>2. Facility manager<br>3. Patient care provider<br>4. Other Specify
4.	Please tell me your highest level of professional qualification.	1. MBBS<br>2. MCPS<br>3. FCPS<br>4. Diploma<br>5. MBA<br>6. BSCN<br>7. MSCN<br>8. MSC<br>9. PhD<br>10. Other Specify
5.	Years of Experience (write in years)	
6.	How long have been working in this facility (write in years)	
7.	What are your main responsibilities?	1. Unit manager<br>2. Facility manager<br>3. Patient care provider<br>4. Other Specify

SECTION 3.1: STAFFING MATERNAL AREA						
S.N	Category of Staff	Total number working in the facility	Sanctioned	Appointed	# of Part Time Staff	Working hours (Morning, evening, night)
a.	Gynecologist/obstetrician					
b.	Anesthetist					
c.	Registrars					
d.	Medical officers					
e.	Matron Nurse					
f.	Registered Nurse					
g.	Midwife					
h.	Nursing assistants					
i.	House officers/interns					
j.	Post graduate Trainees					
k.	Others Specify					
l.	What is nurse to patient ratio in maternal areas?<br>Number of nurses in each maternal area		Labor Room ___________________<br>Maternity wards _________________<br>HDU ___________________			

SECTION 3.2: EQUIPMENTS MATERNAL AREA (Oxygen and Suction)		
a	OBSERVATION: Are there more than one patient assigned per bed?	1. Yes<br>2. No
b	Is oxygen available?	1. Yes<br>2. No
C	What is the source of oxygen in this facility?	1. Central pipe<br>2. Concentrator<br>3. Oxygen cylinder<br>4. Others (specify)
d	How many patients are connected to one source (point/cylinder)<br>oxygen	
e	Is medical air available throughout this facility?<br>(Medical Air is a Combination of Argon, Nitrogen and Oxygen and is<br>Used for OT rooms and anesthesia)	1. Yes<br>2. No

f	Does facility uses humidified oxygen	1. Yes<br>2. No
g	Does the facility have wall suction available?	1. Yes<br>2. No
h	If No: what do you use for suction?	
i	Does maternal units have a functional resuscitation area and crash cart** available?	1. Yes<br>2. No
j	Observation	
k	Have the healthcare providers in maternal unit received training in ADULT RESUSCITATION?	1. Yes (ACLS)<br>2. Yes (BLS)<br>3. No
L	Are there any nutrition services available for mothers in this facility?	1. Yes<br>2. No

**3.2.1. List of Essential equipment**		**Available (Observed)**			**Functionality (Observed)**			**Remarks**
		**Yes**	**No**	**Qty**	**Yes**	**No**	**Qty**	
a.	Vacuum Extractor							
b.	MVA Instruments							
c.	D&E Instrument Kit							
d.	Delivery Instruments kits							
e.	Partograph							
f.	CTG monitors							
g.	Bipap -non=-invasive							
h.	Mechanical Ventilator-							
i.	Physiologic vital monitor for temperature, pulse, respiration, and EKG							
j.	Examination light (flashlight)							
k.	Infant weighting scale							
l.	Blood pressure apparatus and cuffs							
m.	Radiant warmer							
n.	Stethoscope							
o.	Pulse oximeter							
p.	Thermometer							
q.	Glucometer							
r.	Breast pump, manual							
s.	Infusion pumps							

SECTION 3.3: ESSENTIAL AND EMERGENCY MEDICATIONS

**1.**	**MATERNAL UNITS**	**Yes**	**NO**	**QUANTITY IN STOCK**
a.	Metoprolol			
b.	Diazepam			
c.	Diclofenac Sodium			
d.	Hydrocortisone sodium			
e.	Dexamethasone			
f.	Oxytocin			

g.	Lidocaine GEL			
h.	MgSO4			
i.	Lidocaine			
j.	Dobutamine			
k.	Furosemide			
l.	Calcium Gluconate			
m.	Vitamin K			
n.	Water for injection			
o.	Ringer lactate			
p.	Normal Saline			
q.	Dextrose and water 5% (D5W)			
r.	Dextrose and water 10% (D10W)			
s.	Dextrose and water 25% (D25W)			
t.	Epenephrine			
u.	Nor-Epinephrine			

SECTION 3.4: PROTOCOLS/ PATHWAYS (MATERNAL)		
1.	Do you have guideline or pathway for use of oxytocin to prevent post-partum hemorrhage?<br>IF YES, ASK TO SEE THE GUIDELINES.	1. Yes, observed<br>2. Yes, reported, not seen<br>3. No guideline available
2.	Do you have guidelines or pathway on management of pre-term labor?<br>IF YES, ASK TO SEE THE GUIDELINES.	1. Yes, observed<br>2. Yes, reported, not seen<br>3. No guideline available
3.	Do you have guidelines or pathway for use of antibiotics in preterm or prolonged PROM (premature rupture of membranes) to prevent infection	1. Yes, observed<br>2. Yes, reported, not seen<br>3. No guideline available
4.	Do you administer corticosteroids (ACS) to mother with suspected preterm delivery? If yes ask to see guideline?<br><br>Briefly describe ACS criteria	1. Yes, observed<br>2. Yes, reported, not seen<br>3. No guideline available<br>____<br>____
5.	Do you have protocol or pathway for management of pre-eclampsia?<br>IF YES, ASK TO SEE THE GUIDELINES.	1. Yes, observed<br>2. Yes, reported, not seen<br>3. No guideline available
6.	Is hygienic cord care (cut with sterile item and apply disinfectant to tip and stump, and no application of other substances) performed at the facility?	1. Yes<br>2. No
7.	Do you practice delayed cord clamping more than 60 seconds?	1. Yes<br>2. No
8	How do you estimate gestational age of baby?	1. LMP<br>2. Ultrasound<br>3. Physical examination<br>4. Others (specify)
9	Is there a register where delivery/birth information is recorded, i.e., a delivery register?<br>IF YES, ASK TO SEE THE REGISTER	
10	What are immediate first hour (golden minute of life) practices at your facility?<br>Mark all that applies	1. Cord care (cut with sterile item and apply disinfectant to tip and stump, and no application of other substances)<br>2. Dry baby immediately after birth and wrap

		3. Provide Skin to skin contact<br>4. Breastfeeding<br>5. Others (specify)

SECTION 4: NEONATAL SERVICES		
**Details of the Respondent (Details will be required if the respondent is different from the previous section)**		
1.	Age of Respondent (in years)	
2.	Gender of Respondent	1. Male<br>2. Female
3.	What are your main responsibilities?	1. Unit manager<br>2. Facility manager<br>3. Patient care provider<br>4. Other Specify
4.	Please tell me your highest level of professional qualification.	1. MBBS<br>2. MCPS<br>3. FCPS<br>4. Diploma<br>5. MBA<br>6. BSCN<br>7. MSCN<br>8. MSC<br>9. PhD<br>10. Other Specify
5.	Years of Experience (write in years)	
6.	How long have been working in this facility (write in years)	
7.	What are your main responsibilities?	1. Unit manager<br>2. Facility manager<br>3. Patient care provider<br>4. Other Specify

SECTION 4.1: STAFFING NEONATAL AREA

S.N	Category of Staff	Total number working in the facility	Sanctioned	Appointed	# of Part Time Staff	Working hours (Morning, evening, night)
a.	Neonatologist					
b.	Pediatrician					
c.	Pediatric surgeon					

d.	Registrars					
e.	Medical officers					
f.	Matron Nurse					
g.	Registered Nurse					
h.	Midwife					
i.	Nursing assistants					
j.	House officers/interns					
k.	Post graduate Trainees					
l.	Others Specify					
m.	What is nurse to patient ratio in neonatal areas?<br>Number of nurses in each maternal area		Nursery _______________<br>Special care nursery _____________<br>NICU __________________			

SECTION 4.2: EQUIPMENTS NEONATAL AREA (Oxygen and Suction)								
1	OBSERVATION: Are there more than one patient assigned per bed?	1. Yes<br>2. No						
2	Is oxygen available?	1. Yes<br>2. No						
	What is the source of oxygen in this facility?	1. Central pipe<br>2. Concentrator<br>3. Oxygen cylinder<br>4. Others (specify)						
4	How many patients are connected to one source (point/cylinder)<br>oxygen							
3.	Is medical air available throughout this facility?<br>(Medical Air is a Combination of Argon, Nitrogen and Oxygen and is<br>Used for OT rooms and anesthesia)	1. Yes<br>2. No						
4.	Does facility uses humidified oxygen	3. Yes<br>4. No						
7	Does the facility have wall suction available?	3. Yes<br>4. No						
8	If No: what do you use for suction?							
4.2.1. List of Essential equipment		Available (Observed)			Functionality (Observed)			Remarks
		Yes	No	Qty	Yes	No	Qty	
a.	Mechanical Ventilator							
b.	CPAP-							

c.	Cardiac monitors							
d.	High flow							
e.	Illuminator							
f.	Incubators							
g.	Portable x-ray machine							
h.	Warmer							
i.	Apnea monitor							
j.	Phototherapy							
k.	Infusion pumps							
l.	Sterilized Cups and spoons							
m.	Container for storing breastmilk							
n.	Refrigerator for storing breastmilk or drugs							
o.	Milk warmer							
p.	Stethoscope							
q.	Pulse oximeter							
r.	Digital Thermometer							
s.	Mercury Thermometer							
t.	Glucometer							
u.	Suction bulb							
v.	Wall mount suction							
w.	Medical air							
x.	Infant weight scale							
y.	Infant length scale							

SECTION 4.3: ESSENTIAL AND EMERGENCY MEDICATIONS				
		**Yes**	**NO**	**QUANTITY IN STOCK**
a.	Caffeine citrate			
b.	Aminophylin			
c.	Vitamin K			
d.	Ampicilline			
e.	Gentamycine			
f.	Cefotaxime			
g.	Surfactant			
h.	Prostaglandin intravenous / oral			
i.	Phenobarbital			
j.	Levetiracetam -Lerace			
k.	Midazolam			
l.	Diazepam			
m.	Phenytoin			
n.	Epinephrine			
o.	Nor-epenephrine			
p.	Dopamine			
q.	Dexamethasone			
r.	Dextrose and water 5% (D5W)			
s.	Dextrose and water 10% (D10W)			
t.	Dextrose and water 25% (D25W)			
u.	Sodium Chloride (0.9NS)			

4.4: RESUSCITATION		
1.	Does neonatal units have a functional resuscitation area and crash cart** available?	3. Yes<br>4. No
2.	Observation	
3.	Have the health care providers in newborn unit received training in newborn resuscitation?	1. Yes (NRP)<br>2. Yes (HBB)
4.	When was the last training conducted?	

5: CRASH CART SUPPLIES		Available (Observed)			Functionality (Observed)		
		Yes	Number	No	Yes	Number	No
a.	Resuscitation table with heat source						
b.	Defibrillator						
c.	Newborn AMBO Bag WITH and DIFFERENT SIZES MASKS for term and preterm newborns mask						
d.	Laryngoscope available with blade sizes<br>1 (10cm)<br>0 (7.5cm)<br>00(6cm)						
e.	Straight blades<br>Curved blades						
f.	Stylet						
g.	Endotracheal tubes size:<br>2.0<br>2.5<br>3.0<br>3.5<br>4.0						
h.	Electric suction pump (for suction apparatus)						
i.	Capnometer						
j.	Stethoscope						
k.	Insulin Syringes						

6.CRASH CART MEDICATIONS		Yes	Quantity	No
a.	Epinephrine			
b.	Amiodarone			
c.	Atropine			
d.	Magnesium sulfate			
e.	Sodium bicarbonate			
f.	Phenytoin			
g.	Calcium Gluconate			
h.	Dopamine			
i.	Dobutamine			
j.	Nor-Epinephrine			
k.	0.9% N/S			
l.	5% DW			

m.	10% DW			

4.6: PROTOCOLS/ PATHWAYS (NEW BORN AREA)		
1	Do you have guidelines or protocols for newborn admission?<br>IF YES, ASK TO SEE THE GUIDELINES	1. Yes, observed<br>2. Yes, reported, not seen<br>**3.** No guideline available
2	Do you have a guideline or protocol for newborn head to toe assessment?	1. Yes, observed<br>2. Yes, reported, not seen<br>3. No guideline available
3	Where do you record newborn assessment?	
4	Are healthcare providers trained to perform postnatal gestational age assessment (Ballard scoring)	1. Yes<br>2. No
5	Do you have guidelines or protocols for newborn feeding?<br>IF YES, ASK TO SEE THE GUIDELINES.	1. Yes, observed<br>2. Yes, reported, not seen<br>3. No guideline available
6	Do you routinely encourage exclusive use of breastmilk for newborn?	1. Yes<br>2. No
7	Do you have IEC material/leaflets on breastfeeding?	1. Yes<br>2. No
8	Does this facility routinely provide alternative feeding for newborns who cannot breastfeed?	1. Yes<br>2. No
9	Does this facility have a system or means for accessing donor breast milk?	1. Yes<br>2. No
10	Does this facility have formula milk available for newborn?<br>Name of available formula milk?	1. Yes, Name:<br>2. No
11	**OBSERVATION**<br>Record any promotional material you observe on the walls or in corridors?	
12	Do you have guidelines or protocols for newborn thermal management? IF YES, ASK TO SEE THE GUIDELINES	1. Yes, observed<br>2. Yes, reported, not seen<br>3. No guideline available
13	Are healthcare providers trained to provide thermal care (including immediate drying and skin-to-skin care)	1. Yes<br>2. No
14	When was the last training conducted?	
15	Do you have guidelines or protocols for management of Respiratory distress syndrome? IF YES, ASK TO SEE THE GUIDELINES.	1. Yes, observed<br>2. Yes, reported, not seen<br>3. No guideline available
16	Do you have a protocol for managing apnea in preterm babies?	1. Yes, observed<br>2. Yes, reported, not seen<br>3. No guideline available
17	Do you have guidelines or protocols for management of hyperbilirubinemia? IF YES, ASK TO SEE THE GUIDELINES.	1. Yes, observed<br>2. Yes, reported, not seen<br>3. No guideline available
18	Do you have guidelines or protocols for management of asphyxia?<br>IF YES, ASK TO SEE THE GUIDELINES.	1. Yes, observed<br>2. Yes, reported, not seen<br>3. No guideline available

19	Do you have guidelines or protocols for management of sepsis?<br>IF YES, ASK TO SEE THE GUIDELINES.	1. Yes, observed<br>2. Yes, reported, not seen<br>3. No guideline available
20	Do you give prophylactic antibiotics to all preterm newborns?	1. Yes<br>2. No
21	Are there wall mounts of different dosage calculations?	1. Yes take pictures with consent<br>2. No
22	Do you have guidelines or protocols for management of hypoglycemia? IF YES, ASK TO SEE THE GUIDELINES.	1. Yes, observed<br>2. Yes, reported, not seen<br>3. No guideline available
23	How frequently do you monitor blood glucose level?	1. 1 hourly<br>2. 4 hourly<br>3. Once per shift<br>4. Other _____________
24	Do you have a protocol for management of newborns with birth asphyxia? IF YES, ASK TO SEE THE GUIDELINES.	1. Yes, observed<br>2. Yes, reported, not seen<br>3. No guideline available
25	Do you have growth charts in the unit?	1. Yes<br>2. No
**KANGAROO MOTHER CARE**		
2<br>6	Is Kangaroo Mother Care (KMC) for premature/very small babies routinely practiced in this facility?	1. Yes<br>2. No
27	Is there a separate unit with beds for KMC?	1. Yes<br>2. No
28	Is there a register where provision of KMC is recorded?	1. Yes<br>2. No
29	Does the facility has any IEC material/ leaflet or wall mounts on KMC?	1. Yes<br>2. No
**DISCHARGE PATHWAYS and POST NATAL FOLLOW UPS**		
31	Do you have discharge protocol or guidelines?	1. Yes<br>2. No
32	Are there any checklist to assess parental/caregiver competence in providing needed infant care at home (such as ability to feed infant/competency with KMC and routine care)	1. Yes<br>**2.** No
33	When is the first newborn follow-up scheduled after discharge?<br><br>Where do you call them for follow up<br><br>Who examines them at follow up?	
34	Is there a system to ensure adherence for treatment and follow up of newborns/young infants after discharge.	1. Trace through phone calls/text message<br>2. Trace through community volunteers or facility outreach<br>3. Appointed focal point at the facility to manage community follow-up for discharged infants

		4. Identify a community support person for parent<br>5. Use appointment systems<br>6. Use appointment reminder systems<br>7. Identify missed appointments for clinical follow-up and ensuring follow-up<br>8. Other (Specify)
**REFERRAL PATHWAYS**		
35	Do you receive newborn from outside/other facilities?	1. Yes<br>2. No
36	Where do you refer newborns for advance care?	Names and location:
37	Is there a register where out referrals are recorded?	1. Yes<br>2. No
38	Does this facility have access to an ambulance or other vehicle for emergency transport	1. YES (at facility)<br>2. YES (at another facility)<br>3. NO<br>4. Out source
39	Is fuel for the ambulance or other emergency vehicle available? (Enter response by observing)	1. Yes<br>2. No
40	Does the ambulance have oxygen available in it?	1. Yes<br>2. No

**SECTION 5: ANCILLIARY SERVICES AND INFECTION CONTROL**		
**Details of the Respondent (Details will be required if the respondent is different from the previous section)**		
1.	Age of Respondent (in years)	
2.	Gender of Respondent	1. Male<br>2. Female
3.	What are your main responsibilities?	1. Unit manager<br>2. Facility manager<br>3. Patient care provider<br>4. Other Specify
4.	Please tell me your highest level of professional qualification.	1. MBBS<br>2. MCPS<br>3. FCPS<br>4. Diploma<br>5. MBA<br>6. BSCN<br>7. MSCN<br>8. MSC<br>9. PhD<br>10. Other Specify
5.	Years of Experience (write in years)	

6.	How long have been working in this facility (write in years)	
7.	What are your main responsibilities?	1. Unit manager<br>2. Facility manager<br>3. Patient care provider<br>4. Other Specify

SECTION 5.1: STAFFING NEONATAL AREA						
S.N	Category of Staff	Total number working in the facility	Sanctioned	Appointed	# of Part Time Staff	Working hours (Morning, evening, night)
n.	Pharmacist					
o.	Radiologist					
p.	Sonologist					
q.	Technician Radiology					
r.	Technician laboratory					
s.	Technician blood bank					
t.	Phlebotomist					
u.	Nutritionist					
v.	Housekeeping staff					
w.	Untrained technical and ancillary staff					

5.2: ANCILLARY SERVICES		
BLOOD BANK		
1.	Is the blood that is transfused in the facility screened for Hep C ,B ,HIV ,CMV ?	1. Always<br>2. Sometimes<br>3. Rarely<br>4. Never
2.	Does this facility have a refrigerator available and functioning in this service area for the storage of blood?	1. Available and functional<br>2. Available not functional<br>3. Available don’t know if Functioning<br>4. Not available
3.	Is the temperature of the refrigerator monitored at least once every 24 hours?<br>If yes: please ask to see the log used to record the temperature	1. Yes, log Observed<br>2. Yes, log Reported but not seen<br>3. No
4.	Do you have any guidelines on the appropriate use of blood and safe transfusion practices?	1. Yes, observed<br>2. Yes, reported not seen<br>3. No
5.	Have any provider(s) of blood transfusion services received	1. Yes

	any training in the appropriate use of blood and safe transfusion practices in the last two years?	2. No
6.	Are healthcare providers in maternal and newborn units trained for blood transfusion?	1. Yes<br>2. No
7.	When was the last training conducted?	
**RADIOLOGY**		
1.	What radiological services are available in this facility? MARK ALL THAT APPLIES	1. X-ray<br>2. Ultrasound<br>3. CT<br>4. MRI<br>5. ECHO<br>6. Others (specify)

		**Available**			**Functional**		
2.	I would like to know if the following equipment/ items are available and functional today or not available or not functioning today.	Yes	Number	No	Yes	Number	No
a.	X-ray machine						
b.	Portable Xray machine						
c.	Ultrasound equipment						
d.	Portable ultrasound						
e.	CT scan/ MRI						
f.	ECG						
g.	Echocardiogram						

**5.3: INFECTION CONTROL**		
1.	Does this facility have any guidelines on standard precautions for infection prevention? If yes, ask to see the document	1. Yes, observed<br>2. Yes, reported not seen<br>3. No
2.	Are there separte bins for clinical and non-clinical wastes?	1. Yes<br>2. No
3.	Are hand cleaning materials—running water and soap available in the form of sinks at the clinical areas	1) Labor Room<br>i) Yes<br>ii) No<br>2) OR<br>i) Yes<br>ii) No<br>3) Maternal areas<br>i) Yes<br>ii) No<br>4) Nursery<br>i) Yes<br>ii) No<br>5) NICU

		i) Yes<br>ii) No
4.	ARE Sanitizers available at the in-patient and out -patient area?	1.Yes<br>2.No
5.	Are the containers filled?	1.Yes<br>2.No
6.	Are there any wall mounts/ IEC materials placed at unit on hand hygiene practices	1. Yes<br>2. No
7.	Does the facility have regular supply of masks?	1. Yes<br>2. No
8.	What is the policy regarding mask wearing given the Covid-19 pandemic?	1. Everyone should wear mask<br>2. Doctors should wear mask<br>3. OT staff should wear mask<br>4. Others (Specify)

Item		Availability			Functionality		
a.	Electric autoclave (pressure & wet heat)	Yes	No	Qty	Yes	No	Qty
b.	Non-electric autoclave (Functioning through Gas)						
c.	Electric boiler or steamer (no pressure)						
d.	Non-electric pot with cover for boiling/steam						

**Please tell me if the following resources/supplies used for infection control are available in facility?**		
a.	Clean running water (piped, bucket with tap, or pour pitcher)	1. Yes<br>2. No
b.	Hand-washing soap/liquid soap	1. Yes<br>2. No
c.	Alcohol based hand rub	1. Yes<br>2. No
d.	Disposable latex gloves	1. Yes<br>2. No
e.	Waste receptacle (pedal bin) with lid and plastic bin liner	1. Yes<br>2. No
f.	Sharps container ("safety box")	1. Yes<br>2. No
g.	Environmental disinfectant (e.g., chlorine, alcohol)	1. Yes<br>2. No
h.	Disposable syringes with disposable needles	1. Yes<br>2. No
**DISINFECTING FLOOR**		
1.	How frequently is the floor in the unit mopped with water and disinfectant?	1. More than once daily<br>2. Once daily<br>3. Not daily<br>4. No specific schedule
2.	When a newborn is discharged is the cot/incubator wiped down with disinfectant prior to placing another patient in it?	1. Yes, always<br>2. Sometimes, not always<br>3. No
3.	Are blankets and bedding washed between patients?	1. Yes, always<br>2. Sometimes, not always

		3. No
4.	Does the facility have a laundry to wash the blankets and bedding?<br>IF YES, CLARIFY FREQUENCY THAT THE LAUNDRY FUNCTIONS	1. Yes, available daily<br>2. Yes, not available everyday
5.	How often is the diaper disposal container for the unit emptied?	1. More than once daily<br>2. Daily<br>3. When full

**5.4: COMMUNICATIONS**		
1.	Does this facility have a functioning landline telephone?	1. Yes<br>2. No
2.	Does this facility have a functioning mobile telephone?	1. Yes<br>2. No
**5.5: ENVIRONMENT ADDITION OF WASHROOM AT WARD LEVEL**		
1.	Is there any system that allows you to maintain an adequate temperature in maternal and newborn units?	1. Yes, system functional and temperature monitoring possible<br>2. System functional but no temperature monitoring possible<br>3. No system for maintaining adequate temperature
2.	Is there a thermometer or thermostat that shows the room temperature?<br>**NOTE THE CURRENT TEMPERATURE IN THE LABOR ROOM AND POSTNATAL ROOM**	1. Yes<br>2. No<br><br>CENTIGRADE (LABOR ROOM): ______________<br>CENTIGRADE (POST NATAL ROOM): ______________
**5.6: POWER SUPPLY**		
1.	What is the facility's main source of electricity?	1. Central supply of electricity (e.g. National or community grid)<br>2. Generator (fuel or battery operated generator)<br>3. Solar system<br>4. Uninterruptible Power Source
2.	Other than the main or primary source, what is the secondary or backup source of electricity?	1. Central supply of electricity (e.g. National or community grid)<br>2. Generator (fuel or battery operated generator)<br>3. Solar system<br>4. Uninterruptible Power Source<br>5. No secondary source available
**5.7: BASIC CLIENT AMENITIES**		
1.	What is the primary source of water for the facility at this time?	1. Public tap water<br>2.<br>3. Tanker truck<br>4. RO Plant<br>5. No water source
2.	What is the secondary source of water for the facility at this time?	1. Public tap water<br>2. Tanker truck<br>3. RO Plant<br>4. No water source
3.	Is water available from this source on facility premises?	1. Yes, inside the facility<br>2. Yes, within the ground of the facility<br>3. No, outside the facility grounds
4.	Is there a toilet (latrine) on premises in functioning condition that is accessible for general outpatient	1. Yes, gender separated toilets<br>2. Yes, common toilets for all patients

	client use?	3. No, toilet is available<br>4. Toilets Available, but not functional
5.	The facility has whitewashed interior	1. Yes, observed<br>2. No
6.	The facility has Clean exterior	1. Yes, observed<br>2. No
7.	A visible signboard of the facility is present	1. Yes, observed<br>2. No
8.	The facility has client waiting area with ventilation (working fans)	1. Yes, observed<br>2. No

**5.8: HEALTH DATA INDICATORS**		
1.	Do you hold any audit meetings for maternal and newborn mortality or near-misses?	1. Yes, for mothers only<br>2. Yes, for newborns only<br>3. Yes, for both<br>4. No
2.	How frequently are these meeting conducted?	
3.	Do you have register or files where this data is recorded?<br>If yes, take a picture or document last 6 month data	
4.	Do you audit data on Still Birth?<br><br>Do you conduct meetings for still birth s	1. Yes<br>2. No
5.	How frequently are these meeting conducted?	
6.	Do you have register or files where this data is recorded?<br>If yes, take a picture or document last 6 month data	

**GENERAL REMARKS/COMMENTS**

Name and Sign of Data Collector	
Date of Data Collection	
Time when data collection was completed	

Name and Sign of Reviewer	

Appendix S1. Survey Outline

The survey instrument was finalized through a consultative process involving all stakeholders of the national survey. An extensive tool consisting of more than 850 questions/observations/responses was field tested, reviewed, refined and coded where required and printed. The data collection tool comprised of basic information (name, location of health facility), scope of services (maternal, newborn facilities, time of coverage), infrastructure (presence of triage room, labour room, operation theatre, HDU etc.), staffing (separate for maternal and neonatal with appointed and part-time details) and equipment (separately for maternal and newborn areas). Essential and emergency medications were also checked for quantity and in stock details. Resuscitation area was also observed along with crash cart supplies and medications. Questions related to protocols and guidelines for maternal and newborn, KMC, discharge pathways and referral pathways was also included along with observations. Information related to health data indicators, ancillary services and infection control were also included in the study tool. Obstetric and neonatal 21 signal functions were included, adopted from WHO guidelines (Table-1). For the assessment of each signal function, a scoring system was devised as 1.0 for full compliance with associated parameters, 0.5 for partial compliance and 0 (zero) for non-compliance. Cumulative district wise signal functions scores of HCFs were mapped in continual ordinal manner ranging from lowest to highest of 1 (red) to 11(green) for obstetric care signal functions and 1 (red) to 10(green) for neonatal care signal functions for further comparison of services, compliance and analysis. While blank area (white) used for 0 (zero) for no service functions compliance.

All the data collection teams were trained at a central pace in two days training workshops which involve overview of the project, each question of data collection tool followed by a mock activity in the HCF. The team were also trained to observe the availability of equipment, facilities, brochures etc. A booklet covering all the definitions and description of terminology with pictures of instruments was prepared and handed over to all data collectors in hard and soft forms for harmonization of data and to use as ready reference in the field.

Training of field staff was conducted in two phases. First, a two-day master training workshop, including hands-on exercises and mock activities, was convened by senior faculty members from Aga Khan University (AKU) and research team leads from the Health Research Institute (HRI) and the National Institutes of Health (NIH). This was followed by four provincial-level refresher training sessions conducted by senior research team members under the supervision of the lead team to reinforce standardization and address context-specific challenges.

Field data collection was carried out by staff from the Health Research Institute, NIH, who were engaged in line with their primary mandate. These teams were distributed across 12 centres located in major cities throughout the country, with each team comprising four to five members. Each field team included a physician, a public health expert, laboratory personnel, and at least one female member to ensure gender-sensitive engagement.

The survey was completed over a three-month period from July to September 2022. In cases of incomplete data or facility dropouts, revisits were conducted by independent teams during October and November 2022. In addition to routine internal validation by field supervisors, an external team was deployed to assess data completeness, ensure harmonization, and conduct on-the-spot verification. Furthermore, double data entry was performed to enhance accuracy and allow for systematic error correction.

Table S4. Provincial Distribution of Health Workforce and Availability of Delivery Services

	Total<br>n (%)	AJK<br>n (%)	Balochistan<br>n (%)	GB<br>n (%)	KP<br>n (%)	Punjab<br>n (%)	Sindh<br>n (%)
**Total HCFs (N)**	274	19	36	18	58	85	58
**Facilities where births take place**							
	245 (89.4%)	15 (78.9%)	31 (86.1%)	15 (83.3%)	54 (93.1%)	77 (90.6%)	53 (91.4%)
**Facilities with relevant workforce**							
**Gynecologist/ obstetricians**	222 (81.0)	18 (94.7)	17 (47.2)	10 (55.6)	49 (84.5)	79 (92.9)	49 (84.5)
**Neonatologist**	50 (18.5)	0 (0.0)	1 (2.8)	2 (11.1)	20 (35.7)	14 (16.7)	13 (22.4)
**Pediatrician**	209 (77.1)	17 (89.5)	19 (52.8)	11 (61.1)	40 (71.4)	72 (85.7)	50 (86.2)
**Pediatric surgeon**	49 (18.1)	3 (15.8)	3 (8.3)	0 (0.0)	12 (21.4)	22 (26.2)	9 (15.5)
**Nurses***	183 (67.5)	13 (68.4)	7 (19.4)	4 (22.2)	42 (75.0)	73 (86.9)	44 (75.9)

*Nurses include Matron nurses, Registered nurses and nursing assistants in neonatal areas**

Figure S1. Obstetric Signal Function Compliance in Public Health Facilities

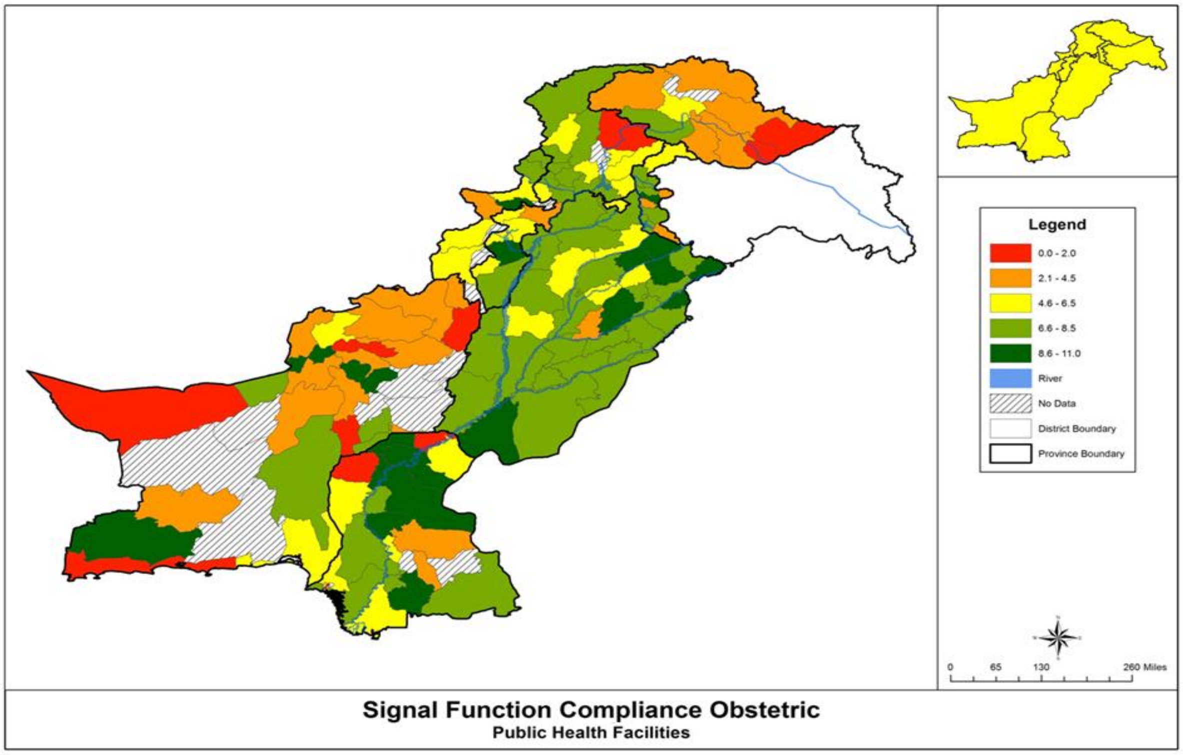

Figure S2. Obstetric Signal Function Compliance in Private Health Facilities

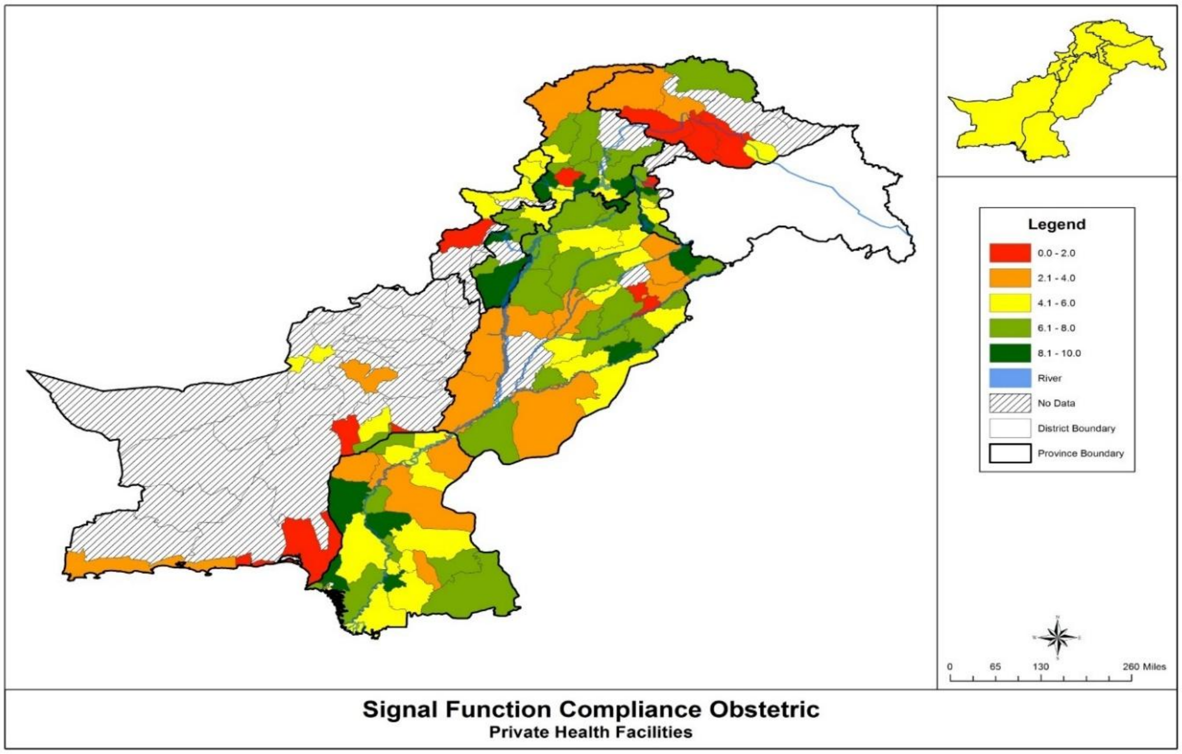

Figure S3. Neonatal Signal Function Compliance in Public Health Facilities

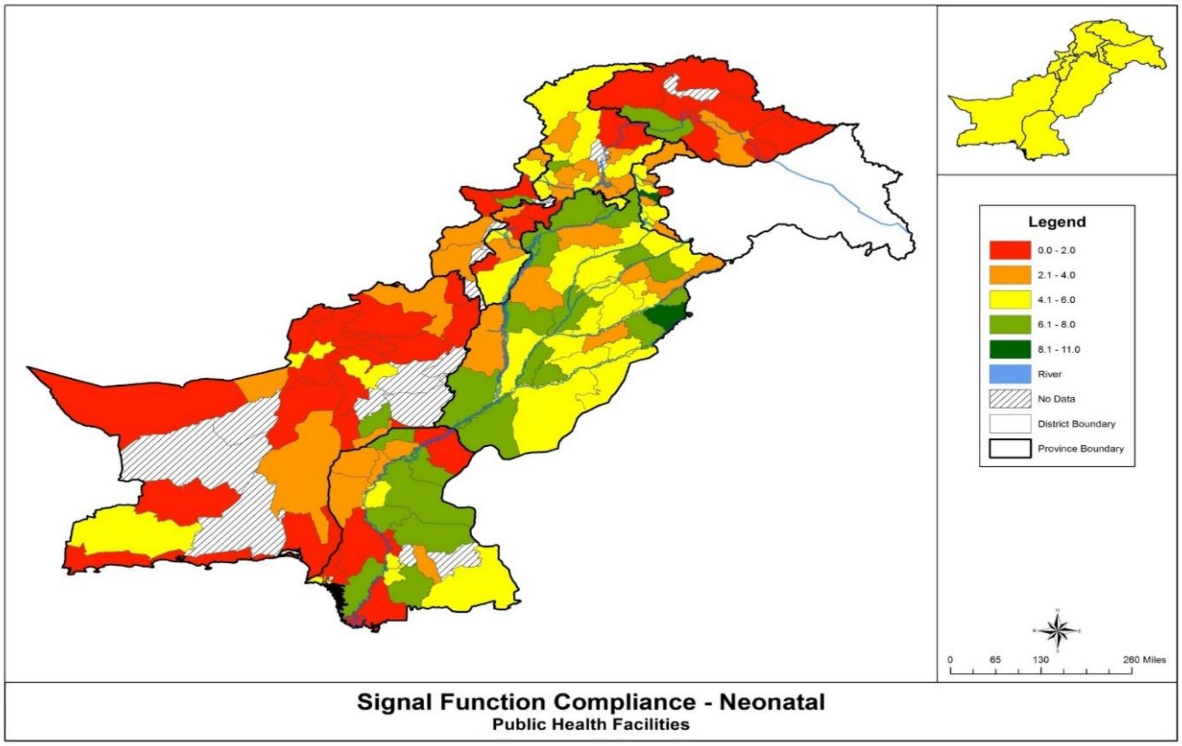

Figure S4. Neonatal Signal Function Compliance in Private Health Facilities

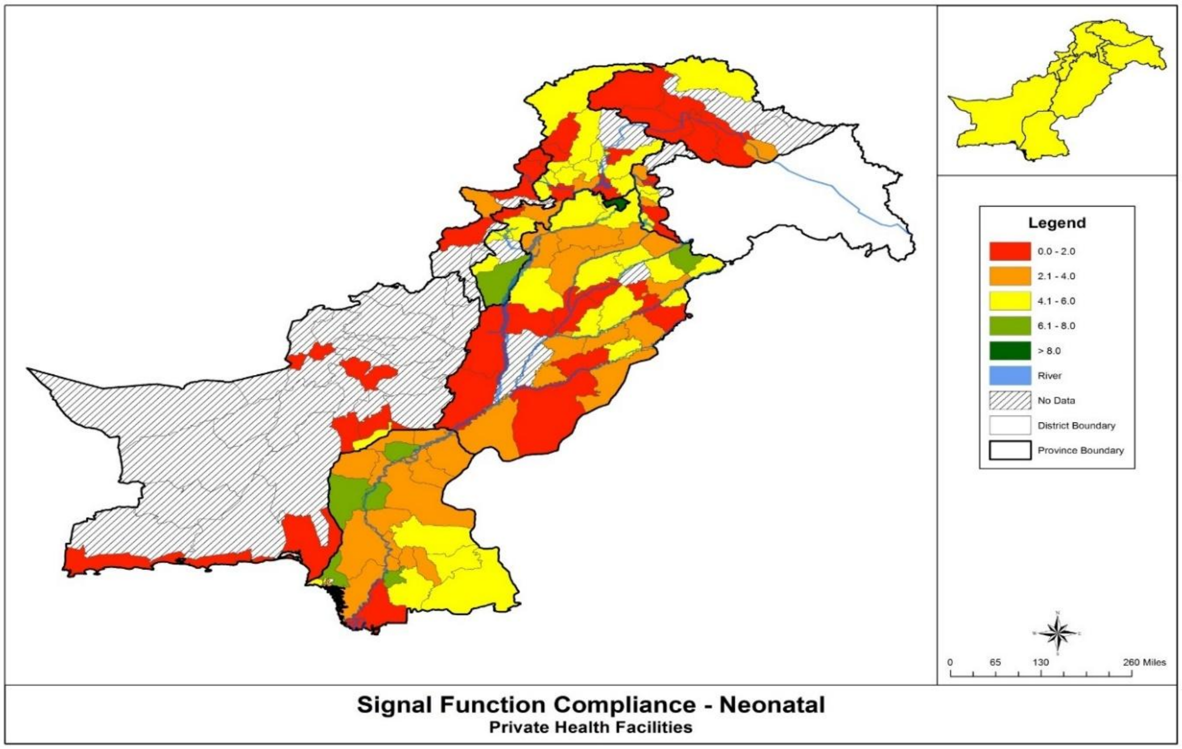

Table S5. Distribution of facilities with resources for infection prevention and management

	Total n (%)	AJK * n (%)	Balochistan n (%)	GB† n (%)	KP‡ n (%)	Punjab n (%)	Sindh n (%)
**Total HCFs^**	**N=274**	**N=19**	**N=36**	**N=18**	**N=58**	**N=85**	**N=58**
**Prevention of Infection**							
Availability of Guidelines	89 (32.5)	5 (26.3)	3 (8.3)	5 (27.8)	6 (10.3)	50 (58.8)	20 (34.5)
Clean running water	259(94.5)	19 (100)	34 (94.4)	17 (94.4)	51 (87.9)	85 (100)	53 (91.4)
Hand-washing soap	238 (86.9)	19 (100)	24 (66.7)	17 (94.4)	45 (77.6)	84 (98.8)	49 (84.5)
Alcohol-based hand rub	170 (62.0)	14 (73.7)	22 (61.1)	9 (50.0)	15 (25.9)	68 (80.0)	42 (72.4)
Disposable latex gloves	246 (89.8)	19 (100)	26 (72.2)	17 (94.4)	50 (86.2)	83 (97.6)	51 (87.9)
Separate bins for clinical and non-clinical wastes	177 (64.6)	13 (68.4)	10 (27.8)	11 (61.1)	31 (53.4)	69 (81.2)	43 (74.1)
Sharps container (safety box)	200/273 (73.3)	18 (94.7)	19 (52.8)	12 (66.7)	33/57 (57.9)	75 (88.2)	43(74.1)
Environmental disinfectant	198 (72.3)	13 (68.4)	24 (66.7)	10 (55.6)	23 (39.7)	76 (89.4)	52(89.7)
Disposable syringes and needles	256/273 (93.8)	18 (94.7)	26 (72.2)	16 (88.9)	56 (96.6)	85 (100.0)	55/57 (96.5)
**Treatment of Severe Infection**							
Availability of Guidelines	30 (10.9)	4 (21.1)	0 (0.0)	0 (0.0)	1 (1.7)	12 (14.1)	13 (22.4)
Availability of Ampicillin	115 (41.8)	8 (42.1)	6 (16.7)	6 (31.6)	32 (55.2)	33 (38.8)	30(51.7)
Availability of Gentamicin	103 (37.5)	8(42.1)	14 (38.9)	6 (31.6)	26 (44.8)	23 (27.1)	26 (44.8)

*Azad Jammu & Kashmir.Gilgit Baltistan.‡Khyber Pakhtunkhwa.^Healthcare Facilities.

Note: Denominators are explicitly stated when they differ from the total sample size (N)
